# Controversies in the Pathogenesis, Diagnosis and Treatment of PCOS: Focus on Insulin Resistance, Inflammation, and Hyperandrogenism

**DOI:** 10.3390/ijms23084110

**Published:** 2022-04-08

**Authors:** Decio Armanini, Marco Boscaro, Luciana Bordin, Chiara Sabbadin

**Affiliations:** 1 Department of Medicine-Endocrinology, University of Padua, 35100 Padua, Italy; marco.boscaro@unipd.it (M.B.); chiarasabbadin.85@gmail.com (C.S.); 2 Department of Molecular Medicine-Biological Chemistry, University of Padua, 35100 Padua, Italy; luciana.bordin@unipd.it

**Keywords:** polycystic ovary syndrome, insulin resistance, aldosterone, hyperandrogenism, metformin, inositol, insulin sensitizers, spironolactone

## Abstract

Polycystic ovary syndrome (PCOS) is a heterogeneous and extremely common disease with symptoms that vary with the age of the patient, typically characterized by hyperandrogenism, chronic oligo-anovulation, and/or several metabolic disorders. The syndrome includes various phenotypes, and the pathogenesis is multifactorial, often involving insulin resistance. This feature is closely related to ovarian dysfunction, inflammation, hyperandrogenism, and metabolic disorders, which characterize and complicate the syndrome. Therapy currently considers both lifestyle improvements and medications, and must be tailored on a case-by-case basis. To date, the published studies have not arrived at a definition of the most suitable therapy for each individual case and many of the drugs used are still off-label. In this review, we discuss some controversial diagnostic and therapeutic aspects of PCOS, such as the role of insulin resistance, inflammation, and hyperandrogenism. We also evaluated the advantages and disadvantages of contraceptive therapy and antiandrogens.

## 1. Introduction

Polycystic ovary syndrome (PCOS) is the most common endocrine disease in women of reproductive age. Its prevalence varies greatly depending on the diagnostic criteria used, from 4–8% according to NIH/NICHD criteria, to approximately 18% according to the Rotterdam criteria [1]. An international evidence-based guideline for the evaluation and management of PCOS more recently endorses and suggests the use of the Rotterdam diagnostic criteria [2]. PCOS is a heterogeneous and extremely common disease, with symptoms that vary with the age of the patient and with therapies that must be tailored on a case-by-case basis. Its typical expression is represented by hyperandrogenism with ovarian dysfunction, chronic oligo-anovulation, and/or micropolycystic morphology of the ovary. Guidelines generally focus on the symptoms of PCOS, but remain more elusive regarding the mechanisms leading to the disease, such as insulin resistance, a very common condition in PCOS, which is worsened by hyperandrogenism-related adipose tissue accumulation [3] and is involved in both the pathogenesis and the progression of the disease [4]. Furthermore, apart from the use of estrogen–progestin, no drug has been approved specifically to counteract metabolic abnormalities and hyperandrogenism, so most drugs are administered off-label.

This review examines some controversies of PCOS related to some important aspects of the disorder: insulin-resistance, inflammation, and hyperandrogenism.

## 2. The Pathogenetic Role of Insulin Resistance in PCOS

Insulin acts as a regulator of glucose homeostasis by stimulating glucose uptake by insulin-sensitive tissues, such as adipose tissue, skeletal muscle, liver, and heart, but also by suppressing hepatic glucose production. Insulin is also able to suppress lipolysis, leading to a decrease in free fatty acid levels, which may mediate insulin’s action on hepatic glucose production. Insulin resistance is defined as a decreased ability of insulin to carry out these metabolic actions inherent in glucose uptake and production and lipolysis, thus leading to compensatory high insulin levels, both at baseline and after glucose loading, if pancreatic function is normal. There is still no consensus on the exact mechanism that leads to insulin resistance in PCOS, regardless of body mass index (BMI). An old study argued that in PCOS, the mechanism underlying insulin resistance decreased autophosphorylation of the insulin receptor following insulin binding [5].

**Figure 1:**
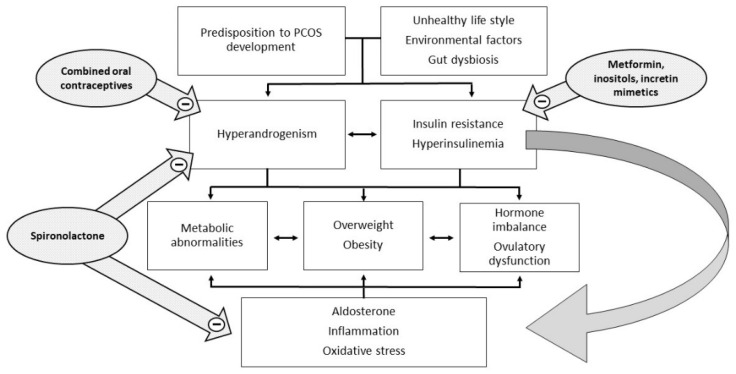
Pathophysiology and potential therapeutic targets of PCOS.

The mechanisms by which insulin resistance exerts its effects have only recently been well described [6]. At a liver and skeletal muscle level, insulin resistance increases lipolysis with the accumulation of non-esterified fatty acids. The accumulation of intrahepatic lipids activates the diacylglycerol/protein kinase C axis and inhibits the insulin receptor, also affecting insulin signaling and subsequent gluconeogenesis. In skeletal muscle, the inhibition of phosphoinositide-3 kinase and phosphorylation of insulin receptor substrate 1 leads to impaired insulin signaling by altering the GLUT-4 expression and glucose uptake [6,7]. The consequence of hyperinsulinemia compensatory to insulin resistance is an overstimulation of non-insulin-sensitive tissues, such as the ovaries. In particular, insulin and LH act synergistically on the theca cells, stimulating ovarian androgen production [4]. In addition, insulin acts both directly as a co-gonadotropin, enhancing LH activity by stimulating the expression of receptors for LH, insulin, and IGF on granulosa cells, and indirectly by impairing the regulation of the hypothalamic–pituitary–ovarian axis. Hyperinsulinemia increases the adrenal steroid response to ACTH stimuli and decreases the synthesis of sex hormone binding globulin (SHBG) in the liver, with a consequent increase of both total and free androgen levels (Figure 1).

Recent studies have hypothesized the role of the gut microbiome as a cause or effect of BMI, insulin resistance, and inflammation in PCOS. Gut dysbiosis due to poor-quality diet could cause the passage of lipopolysaccharides produced by Gram negative micro-organisms into the circulation. The consequence could be the activation of the immune system, insulin resistance, and hyperandrogenism [8]. A recent revision of 31 studies published in the last 10 years reported reduced alpha diversity and dysbiosis in women with PCOS [9]. Treatment of PCOS with prebiotics, probiotics, and synbiotics could have some beneficial effects on metabolic and biochemical profiles. Further studies should investigate the role of the microbiome in the pathogenesis and management of PCOS.

## 3. The Pathogenetic Role of Inflammation in PCOS

PCOS has also been associated with chronic low-grade inflammation, characterized by increased white blood cell count, high levels of C-reactive protein (CRP), interleukin 6 (IL-6), interleukin 18 (IL-18), monocyte chemoattractant protein-1, and macrophage inflammatory protein-1. Insulin resistance is related to inflammation. For example, an exaggerated production of tumor necrosis factor (TNF-α) produced by monocytes as a response to hyperglycemia could exacerbate the metabolic and hormonal abnormalities of PCOS [10]. Recently, advanced glycosylation end products (AGEs) and their receptors implicated in the inflammation and oxidative stress cascades have also been found to be overexpressed in PCOS women [11]. The release of inflammatory markers is associated with long-term metabolic complications and high cardiovascular risk [12].

An underestimated factor in the diagnosis and treatment of PCOS is aldosterone [13]. It has been shown that aldosterone and, in particular, the aldosterone/renin ratio are often increased in PCOS, and this accentuates the underlying inflammatory state and might be involved in the development of some metabolic and cardiovascular disorders [14]. We characterized aldosterone receptors in human mononuclear leukocytes [15], and subsequent studies confirmed that the incubation of lymphocytes with excess aldosterone increased the protein expression of PAI 1 and p22phox, two markers of inflammation [16]. Many studies have reported the role of mineralocorticoid receptor blockers, such as spironolactone, not only in the treatment of hyperaldosteronism and resistant hypertension, but also in the prevention of metabolic and cardiovascular complications and cerebrovascular accidents in patients with normal values of aldosterone [17].

Another important pro-inflammatory agent involved in the pathogenesis of PCOS is adipose tissue [18]. It is known that adipose tissue-resident macrophages release TNF-*α* and IL-6, which are implicated in the induction of insulin resistance [19]. Hyperandrogenism leads to aberrant adipose tissue functions in PCOS [20]. Insulin resistance, hyperandrogenism, chronic low-grade inflammation, and adipose tissue hypertrophy and dysfunction may act together in a vicious cycle in the pathophysiology of PCOS. These observations need confirmation in larger studies directly assessing the presence of inflammation in the fat tissues of PCOS women [21].

## 4. The Pathogenetic Role of Hyperandrogenism in PCOS

Hyperandrogenism in PCOS could be caused by defective intrinsic steroidogenesis in ovarian theca cells [11] or by elevated LH levels due to altered regulation of the hypothalamic–pituitary axis, also influenced by insulin. Alterations in adrenal steroidogenesis due to CYP17α1 hyperactivation [22] could also contribute to the hyperandrogenism of PCOS [23]. Increased peripheral cortisol metabolism has also been proposed as a contributor to adrenal hyperandrogenism. Reduced cortisol levels cause inadequate negative feedback on the hypothalamic–pituitary–adrenal axis with increased pituitary ACTH synthesis and stimulation of adrenal steroidogenesis [24]. If insulin resistance leads to hyperandrogenism and anovulation, hyperandrogenism is also recognized as one of the possible causes of insulin resistance in PCOS (Figure 1). Androgen excess during intrauterine life or in the immediate post-natal period has been shown to accentuate visceral adiposity and insulin resistance. Hyperandrogenic PCOS phenotypes show an increased level of insulin resistance and metabolic complications [25]. The administration of drugs with anti-androgenic activity improves insulin resistance [26]. At the level of adipose tissue, testosterone acts by decreasing protein kinase C (PKC) phosphorylation [27], whereas on skeletal muscle, it acts by increasing the phosphorylation of the mammalian target of rapamycin (mTOR) and ribosomal kinase S6 (S6K), leading to increased serine phosphorylation of IRS-1 [28]. These two androgen-mediated mechanisms exacerbate insulin resistance in adipose tissue and skeletal muscle, respectively.

## 5. Controversies in the Diagnosis of PCOS

Beyond the multifactorial physiopathology of PCOS, the diagnosis can also be complex, firstly because of the presence of different diagnostic criteria and then because of the controversies surrounding the definition and evaluation of the typical features of PCOS, which are oligo-anovulation, clinical and/or biochemical hyperandrogenism, and polycystic ovarian morphology [29]. The Rotterdam criteria (2003), more recently endorsed by international evidence-based guideline [2], requires the presence of at least two of the three criteria to diagnose PCOS in adults, after excluding other causes that could mimic PCOS, such as, in particular, late-onset congenital adrenal hyperplasia and hyperprolactinemia. Based on the presence or absence of these three criteria, the guidelines distinguish four distinct phenotypes by the presence of all criteria for phenotype (A), presence of androgen excess and oligo-anovulation for phenotype (B), and presence of androgen excess and polycystic ovarian morphology for (C). Phenotype (D), only with polycystic ovarian morphology and oligo-anovulation, is also considered among the forms of PCOS [30]. However, this latter phenotype is not included among PCOS according to the Androgen Excess Society guideline, which suggests the presence of both hyperandrogenism and ovarian dysfunction [31].

Increasing evidence suggests the importance of this phenotypic division in order to better understand the pathophysiology of PCOS, and to predict adverse reproductive, metabolic, and cardiovascular outcomes. Previous studies have associated the androgenic phenotype with more severe metabolic alterations [32,33]. In contrast, normo-androgenic PCOS (phenotype D) have a lower risk of metabolic disorders [25].

Dapas et al. recently analyzed the heterogeneity of PCOS phenotypes by using biochemical and genotypic data from a published PCOS genome-wide association study [34]. Clustering revealed two major distinct PCOS subtypes: a “reproductive” group characterized by high LH and SHBG levels, but with normal/low BMI and normal insulin levels, and a “metabolic” group with high BMI, glucose and insulin levels, but with low LH and SHBG levels. In this study, the authors identified alleles at four loci associated with the “reproductive” subtype and one locus significantly associated with the “metabolic” subtype. However, the clinical picture could also be influenced by age, ethnicity, and environmental factors.

Another challenging step in the diagnosis of PCOS is the assessment of hyperandrogenism. Androstenedione is the main androgen produced by ovaries and it is converted to testosterone in peripheral tissues by the enzyme 17β-hydroxysteroid dehydrogenase. It has a lower affinity for androgen receptor than testosterone and dihydrotestosterone (DHT). Testosterone is usually measured in PCOS, however, the common direct assays, such as RIA, ELISA, and CLIA, show poor sensitivity, mainly because they were designed to measure testosterone in males [29]. Mass spectrometry after extraction and liquid or gas chromatography has the highest accuracy, but is expensive, is not routinely used, and standardized [35]. As direct assays to measure free testosterone are not entirely reliable, another useful tool for the assessment of biochemical hyperandrogenism is free testosterone calculated using the formula of Vermeulen and collogues [36]. Serum free testosterone is higher than total testosterone, especially in PCOS, who commonly have reduced SHBG levels, mainly due to insulin resistance. However, the assessment could be biased by inaccurate measurement of SHBG [35]. There are other circulating (11-oxygenated) androgens secreted mainly by the adrenals and converted to active hormones (11-ketotestosterone and 11-ketodihydrotestosterone) [37]. It is noteworthy that in PCOS, the serum 11-ketotestosterone has been found to be three to five times higher than the testosterone.

The assessment of insulin resistance is another major problem for clinicians. The first problem is related to the wide variability in insulin sensitivity between subjects, some of who show a common overlap of insulin levels like those of insulin resistance. When the mechanisms of insulin function are considered to be quantitative or continuous variables from an evolutionary perspective, it is likely that all women with PCOS, whether obese or lean, have insulin resistance [38]. Previous studies found that PCOS women have a significant reduction in insulin sensitivity compared to BMI and age-matched controls [25,39]. In evolutionary terms, women with a PCOS metabolic phenotype would have increased survival chances during times of environmental or physiological need for altered energy metabolism, but would be more vulnerable to the pathological effects of insulin resistance when exposed to modern lifestyle factors [38].

The second problem is related to the various methods of measurement (fasting insulin or after glucose loading), which do not correlate with what has been found using more accurate and sophisticated laboratory methods [40]. The most reliable technique for measuring insulin resistance should be the hyperinsulinemic euglycemic clamp. Unfortunately, this method is used very little in clinical practice due to a lack of practicality. In clinical practice, HOMA-IR is the most common test used in research studies to measure insulin resistance, as it has been shown to closely approximate the results of clamp studies over many years [41,42]. Indeed, in women with PCOS, these surrogate indexes revealed fair correlations with direct measures of in vivo insulin action. In actual fact, they are more valuable in ruling in rather than ruling out insulin resistance in PCOS, especially among normal weight women [43]. Insulin levels, which mainly drive these surrogate indexes, depend on several factors, not only insulin sensitivity and glucose levels. In particular, obesity, age, androgen levels, metabolism and clearance of insulin, and enzymatic degradation of the insulin–receptor complex are perhaps more valuable than insulin itself [44].

## 6. Controversies in the Treatment of PCOS

PCOS is a clinical heterogeneous condition and apart from its classic symptoms including infertility and hyperandrogenic disorders, it can be associated with several cardiovascular comorbidities, such as insulin resistance, impaired glucose tolerance, dyslipidemia, obesity, and hypertension [14]. The approach to the management of PCOS should consider not only patients’ demands, but also related comorbidities. Insulin resistance and hyperandrogenism are the most typical features of the syndrome, and their close relationship influences not only reproductive function, but also the metabolic profile of PCOS patients, regardless of BMI [45].

Even if insulin resistance does not represent one of the diagnostic criteria, it is one of the main targets of PCOS therapy together with combined oral contraceptives (COCs) [46]. Physical activity and diet represent the first therapeutic approach for PCOS patients, improving weight, insulin resistance, hyperandrogenism, and the inflammatory state often present in PCOS [47]. However, different medical therapy options can be considered when lifestyle changes are not sufficient to improve reproductive and/or metabolic function and when patients complain hyperandrogenic skin disorders [48].

### 6.1. Insulin Sensitizers

Among all of the insulin sensitizers, metformin is the most widely used in PCOS, due to its efficacy and safety. It is indicated in combination with COCs, especially for overweight or obese patients, or when COCs are contraindicated [46]. However, the beneficial effects of metformin are increasingly evident, especially when combined with lifestyle modifications, improving the pathogenetic mechanisms underlying PCOS, restoring ovarian function, and improving metabolic profile, especially insulin sensitivity and, unlike COCs, lipoprotein pattern [49]. Recent studies have shown that metformin can improve the inflammatory state both indirectly by improving metabolic parameters, and directly through its anti-inflammatory effect. This mechanism can regulate T-cell balance as reported in mice, where metformin reduces CD4 +/IL-17 + (TH17) and increases CD4 +/Foxp3 + (TREG) [50]. In addition, metformin performs its beneficial anti-inflammatory and health-promoting actions by influencing other structures, such as mitochondrial function, modulation of immunity through mechanisms not yet fully understood, and independently of its role in glycemic control. Another often forgotten aspect concerns depression and other psychiatric disorders, which affect PCOS patients. Metformin has been demonstrated to improve several mental health disorders, indirectly through the improvement of some hormonal and metabolic aspects [51], and directly through its action on the synthesis and secretion of neurosteroids, such as allopregnanolone [52].

Along the same lines, in the last years, several studies have reported the beneficial effects of inositol supplementation in improving the metabolic and hyperandrogenic profile of PCOS women [53]. Myo-inositol (MYO) and D-chiro-inositol (DCI) are the most abundant forms present in humans, primarily derived from a dietary source. MYO can be converted to DCI by an epimerase enzyme, stimulated by insulin. As a result, the DCI/MYO ratio is regulated by every organ and tissue, and is involved in several physiological processes, such as ion channel permeability, cytoskeleton remodeling, developmental processes, stress response, and endocrine signal transduction. In particular, several studies have confirmed the role of MYO and DCI as insulin sensitizers, improving both metabolic and oxidative imbalance [54]. In addition, in the ovary, MYO also acts as a second messenger of FSH signaling: its supplementation can restore regular menses and ovulation, improve oocyte and embryo quality, and reduce the amount of recombinant FSH administered during ovarian stimulation protocols. On the contrary, the supplementation of DCI alone may worsen the insulin-mediated androgen synthesis and fertility in PCOS women [53]. Subsequent studies have demonstrated that the combination MYO and DCI in a 40:1 ratio is the most similar to the plasma ratio reported in healthy women and is the most effective at improving ovarian function and metabolic profile in PCOS patients [55]. Finally, the association of MYO and/or DCI with metformin and/or COCs could have a synergistic action and reduce adverse effects [48].

Recent studies have also evaluated the effects of other insulin sensitizer drugs, well-known and prescribed for diabetes mellitus type 2, such as incretin mimetics and sodium–glucose co-transporter type 2 inhibitors (SGLT2-i), which could be probably more effective on certain comorbidities, such as obesity and cardiovascular system [48].

In particular, among incretin mimetics, glucagon-like peptide-1 receptor agonists (GLP-1 RA) offer a great opportunity to simultaneously treat different conditions of PCOS, improving body weight, insulin resistance, and cardiovascular risk. In the last years, several studies demonstrated safety and efficacy of exenatide and liraglutide use, alone or in combinations with metformin, in PCOS women [56]. The combination of GLP-1 RA and metformin seems to be superior to each single-agent alone, for improving not only BMI, central adiposity, and insulin resistance, but also menstrual cycles and hyperandrogenism, especially in obese PCOS patients [57,58].

SGLT2-i improves the metabolic profile in patients with diabetes increasing glycosuria by inhibiting glucose reuptake in the renal proximal tubule. As a result, they induce a reduction of both arterial pressure and body weight, demonstrating impressive cardio- and nephro-protective effects [59]. Recent studies have shown that the use of SGLT2-i improves anthropometric parameters and body composition in PCOS patients, but the effects on ovulation, menstrual frequency, and hyperandrogenism have not been assessed yet [60].

Finally, emerging data support the beneficial effects of these antidiabetic drugs on gut dysbiosis, which could play a pathogenetic role in PCOS [8]. In particular, the most studied medication is metformin, which seems to positively influence microbiota diversity and composition [61].

It is important to stress that all of these antidiabetic agents are still not approved for the treatment of PCOS. More randomized-control trials and long-term studies are needed to evaluate the beneficial effects of these drugs, which could be an attractive therapeutic tool in the management of PCOS.

### 6.2. Hormonal Contraceptives

Hormonal contraceptives, whether oral, patch, or vaginal, usually contain an estrogen ethinyl estradiol or estradiol valerate and a progestin. Their action is mediated by suppressing gonadotropin secretion and consequently ovarian androgen secretion, increasing SHBG synthesis and reducing the total and free circulating androgens (Figure 1). In PCOS women, COCs can improve the clinical and biochemical picture of hyperandrogenism and some metabolic aspects such as alterations in cholesterol and fat distribution [62], but they can also cause side effects that must be considered, especially in PCOS women with metabolic and cardiovascular risk factors [63]. The excess estrogenic component may worsen insulin resistance, but its effect is modulated by the associated progestin [64]. Ideally, a progestin with the lowest possible androgenic action such as cyproterone acetate, chlormalidone, and drospirenone should be used. However, they have been associated with increased risk of venous thrombosis [65,66,67].

COCs also exacerbate the inflammatory state present in these patients, which is likely related to increased aldosterone–renin ratio and CRP [12,14]. It is known that aldosterone is the main proinflammatory hormone and, unfortunately, the use of COCs stimulates the synthesis of angiotensinogen and activates the renin–angiotensin–aldosterone system (RAAS) [13,14,68]. The consequence is fluid retention frequently complained by these patients, hypertension (which is more frequent in PCOS women), the accentuation of insulin resistance, weight gain, and depression. These disorders are uncommon in non-insulin resistant PCOS women or in non-PCOS women. These side effects are also accentuated by the effect of estrogen to increase cortisol binding globulin (CBG) and stimulate ACTH synthesis to maintain adequate free cortisol [24]. The relative increase in ACTH leads to an increase in adrenal androgens produced by the reticularis zone. It is important to keep in mind that previous studies have also demonstrated an adrenal contribution to hyperandrogenism in PCOS [69]. COCs should be used with caution in PCOS patients with pre-existing metabolic and cardiovascular risk factor, and monitored during follow-up for the occurrence of possible related side effects.

Another frequent adverse consequence of long-term contraceptive therapy is amenorrhea upon discontinuation of the contraceptive. It is well known that the positive effect of COCs on hyperandrogenism requires an adequate duration of therapy, and in a significant amount of cases, post-contraceptive hypothalamic amenorrhea occurs. This side effect often resolves after a few months, but in some cases, it persists. The risk of amenorrhea on discontinuation is accentuated in PCOS patients who already had oligo- or amenorrhea before therapy.

Third-generation contraceptives containing less estrogen have been studied and are effective on signs of hyperandrogenism, but several studies have raised serious questions about long-term cardio-metabolic effects. Unfortunately, the available studies refer only to short-term evaluations and the results are discordant [48]. In addition, these studies are done on a limited number of patients, and important factors that may lead to confusion in interpretation are often not considered. There is an urgent need to perform adequate studies as number of participants and as criteria for metabolic and cardiovascular risk assessment.

The recent development of two COCs containing natural estrogen (17-estradiol and 17-estradiol valerate) could be a valid therapeutic alternative in PCOS, showing a more favorable effect on metabolic profile, RAAS induction, and hemostatic balance compared with ethinyl estradiol [70,71]. These COCs also need validation after more prolonged administration.

Contraceptives containing only progestins are also commercially available, but in PCOS patients, they are not helpful for treating the signs and symptoms related to hyperandrogenism.

### 6.3. Antiandrogens

The most used antiandrogens are cyproterone acetate, spironolactone, finasteride, and flutamide. Thus, all the proposed drugs have an antiandrogenic action, but the possible side effects of each of the therapies are frequently overlooked.

Cyproterone acetate is a potent antiandrogen progestin: it is also a component of a formulation of the trade associated with ethinyl estradiol, but often, to have a sufficient effect, it is necessary to add an additional dose of the drug in the first half of the cycle. Its prescription should be carefully considered because of the risk not only of venous thrombosis, but also of meningioma, which show a strong and dose-dependent association with cyproterone acetate [72].

Spironolactone has several antiandrogen effects such as androgen receptor blockade, aromatase stimulation, and direct effect of partial blockade of androgen synthesis, not only in the gonads but also in the adrenals through a direct effect on ovarian and adrenal androgen synthesis [26,73]. It has an excellent effect on acne that regresses in more than 90% of cases if used for an adequate period of at least 9 months to a year, unlike the pill, which almost always does not maintain the effect when discontinued, even if used for a very long time. The effect on hair loss occurs over a longer period of time because the half-life of hair is very long and therefore at least 5–6 months of therapy are required to see a subjective effect [26]. In addition, spironolactone is a mineralocorticoid receptor antagonist and its use as monotherapy or in addition to COCs has been proposed to reduce side effects related to RAAS activation in PCOS, particularly the pro-inflammatory status, some metabolic alterations, and hypertension [74]. The adverse effects are intermenstrual bleedings, related to a selective progesterone receptor modulator effect of spironolactone [75]. This disorder can be treated with the addition of progestin at mid-cycle for a limited period in order to allow the endogenous progesterone to activate adequately. The addition of pure licorice has been proposed to regulate the hyperkalemic and hypotensive effect of spironolactone in normotensive women, and to enhance the antiandrogenic effects by partially blocking 17β-hydroxystreroid dehydrogenase in the adrenals and ovaries [76,77]. An important issue to consider with the patient is that spironolactone should not be used in women with a risk of pregnancy and therefore should always be associated with condom use. Many couples accept this solution, especially in cases where the pill has given side effects that induced the patient to exclude further use.

Finasteride and dutasteride block the 5 alpha reductase and thus reduce the effect of androgens by blocking their activation to DHT [78]. Recently, this therapy has been associated with major disorders, and some authors have highlighted post-finasteride syndrome because the drug would also act on brain neurosteroids, which are important for proper functioning of certain centers in the cerebral cortex. Finasteride is also typically used alone or combined with estrogens in cases of significant hair loss. 

Flutamide has an excellent androgen receptor blocking action, but, in some cases, can give major effects, including fulminant hepatitis [79,80].

Considering the positive and negative effects of various antiandrogens, spironolactone should be preferred as it also has a potent anti-inflammatory action. The issue of association between spironolactone and COCs is also debated, especially in cases where the couple does not use condoms. Spironolactone counteracts many potentially negative effects of the pill, such as fluid retention and insulin resistance, but it does not counteract the risk of amenorrhea upon discontinuation.

## 7. Conclusions

We have reviewed some of the controversies related to insulin resistance, inflammation, and hyperandrogenism in the pathogenesis, diagnosis, and treatment of PCOS. The diagnostic and therapeutic definition has been revised and agreed upon in the recent international guidelines [2]. These include 166 recommendations and were compiled by 3000 health professionals from 71 countries. They are probably one of the most comprehensive set of guidelines every produced, and agreed on, in any specialty.

Therapy must be individual, because the syndrome has various phenotypes. It is essential to listen to the patient in order to try to remedy aesthetic problems, considering the possible metabolic and cardiovascular implications that could be already present or influenced by dietary habits, lifestyle, and therapy. The ideal therapy should try to improve and solve the problems presented by the patients, and consider the positive or negative long-term effects. It is important to inform patients of these prospects. We have mentioned some new therapies that could be promising, such as insulin sensitizers other than metformin. Excluding COCs, all the other mentioned drugs for PCOS are still off-label. Further studies in larger samples are needed to evaluate the efficacy of these new therapeutic approaches, as well as to prevent future metabolic and cardiovascular disorders.

The treatment of this syndrome remains complex and certainly the interrelationship between genetics, insulin resistance, and inflammation must be the subject of new studies. The recent international guidelines have recommended that lifestyle-based therapies should be the first-line of treatment for all women with PCOS. This review has highlighted the importance of a variety of medications that could have a synergistic effect on multiple features of this syndrome, when used as an adjunct to lifestyle-based therapies.

## Data Availability

Not applicable.
